# Psychological Distress and Protective Behaviors During the COVID-19 Pandemic Among Different Populations: Hong Kong General Population, Taiwan Healthcare Workers, and Taiwan Outpatients

**DOI:** 10.3389/fmed.2022.800962

**Published:** 2022-02-15

**Authors:** Gary Ka-Ki Chung, Carol Strong, Yat-Hang Chan, Roger Yat-Nork Chung, Jung-Sheng Chen, Yi-Hsuan Lin, Ru-Yi Huang, Chung-Ying Lin, Nai-Ying Ko

**Affiliations:** ^1^CUHK Institute of Health Equity, The Chinese University of Hong Kong, Shatin, Hong Kong SAR, China; ^2^Department of Public Health, College of Medicine, National Cheng Kung University, Tainan, Taiwan; ^3^The Jockey Club School of Public Health and Primary Care, The Chinese University of Hong Kong, Shatin, Hong Kong SAR, China; ^4^Department of Medical Research, E-Da Hospital, Kaohsiung, Taiwan; ^5^Department of Nursing, College of Medicine, National Cheng Kung University, Tainan, Taiwan; ^6^Department of Family and Community Medicine, E-Da Hospital, Kaohsiung, Taiwan; ^7^School of Medicine for International Students, College of Medicine, I-Shou University, Kaohsiung, Taiwan; ^8^Institute of Allied Health Sciences, College of Medicine, National Cheng Kung University, Tainan, Taiwan; ^9^Department of Occupational Therapy, College of Medicine, National Cheng Kung University, Tainan, Taiwan; ^10^Biostatistics Consulting Center, National Cheng Kung University Hospital, College of Medicine, National Cheng Kung University, Tainan, Taiwan

**Keywords:** Asia, COVID-19, infection, psychological distress, protective behavior

## Abstract

**Purpose:**

The novel coronavirus disease 2019 (COVID-19) caused psychological distress and changed human living styles. However, rare studies have examined the psychological distress and protective behaviors across different populations. Therefore, the present study aimed to assess psychological distress, protective behaviors, and potential predictors of psychological distress and protective behaviors across the Hong Kong general population, Taiwan healthcare workers, and Taiwan outpatients.

**Methods:**

A cross-sectional design was used to recruit participants from Hong Kong and Taiwan. Telephone interviews were carried out for Hong Kong participants (*n* = 1,067; 30.2% male participants); online surveys were used for Taiwan healthcare workers (*n* = 500; 8.0% male participants) and Taiwan outpatients (*n* = 192; 32.8% male participants). All the participants completed questions on psychological distress and protective behaviors. Multiple linear regressions and multivariable logistic regressions were employed to explore the potential predictors of psychological distress and protective behaviors, respectively.

**Results:**

Hong Kong participants had significantly lower levels of psychological distress than Taiwan participants [mean (SD) = 0.16 (0.39) vs. 0.47 (0.59) in healthcare workers and 0.46 (0.65) in outpatients; *p* < 0.001]. Hong Kong participants (51.7%) and Taiwan outpatients had more people showing fear of COVID-19 (52.0%) than Taiwan healthcare providers (40.8%; *p* < 0.001). Moreover, Hong Kong participants engaged the most in protective behaviors, followed by Taiwan healthcare providers and Taiwan outpatients (*p* < 0.001). Moreover, being a female, fear of COVID-19 and worry about personal savings were associated with protective behaviors in general.

**Conclusions:**

Despite the greater COVID-19 severity and fear of COVID-19 in Hong Kong, the general population in Hong Kong experienced less psychosocial distress with higher compliance to protective behaviors than the other groups in Taiwan.

## Introduction

The long-lasting infection disease, novel coronavirus disease 2019 (COVID-19), has been announced as a global pandemic by the World Health Organization (WHO) (1) over one and a half years. However, the severity and infection rate of COVID-19 remains high worldwide with the emergence of several COVID-19 virus variants (2, 3). Although COVID-19 vaccines have been rapidly developed and implemented (4), concerns over vaccine uptake (5–8) and their efficacy against the emerging COVID-19 variants (9, 10) may hamper the progress toward herd immunity and recovery from COVID-19. Consequently, the psychological distress caused by the COVID-19 (11–14) is unlikely to be resolved in a short period of time. Indeed, several estimations suggest that the virus will not be under control shortly (15, 16). Therefore, the experience from the early stage of COVID-19 pandemic regarding psychological distress (i.e., unpleasant feelings and perceived discomforts, such as depression and anxiety) (17) and protective behaviors would be of great value to assist policymakers and relevant stakeholders in taking care of their citizens under the current or future COVID-19 pandemic.

Several studies have examined the psychological distress (especially in anxiety and depression) during the COVID-19 pandemic (18–29). Specifically, Ahorsu et al. (18) and Pramukti et al. (26) found that university students have issues of anxiety and suicidal ideation during the COVID-19 pandemic. Hasannia et al. (20), Patel et al. (24), and Patil et al. (25) found that healthcare workers have issues of psychological distress related to being stigmatized, burnout, and anxiety. The psychological distress issues among healthcare workers have been summarized by Olashore et al. (23) using a systematic review that the prevalence rates of anxiety (9.5–73.3%) and depression (12.5–71.9%) were high among healthcare workers in African countries. Regarding the general population, Mamun et al. (22) found that the Bangladeshi general population had a relatively high rate of suicidal ideation (5–33%), and depression is one of the key factors explaining suicidal ideation. Moreover, Alimoradi et al. (19) conducted a systematic review with meta-analysis and found that fear of COVID-19 was moderately associated with psychological distress.

We have identified three important populations across two regions (i.e., Hong Kong and Taiwan) sharing similar subcultures but with different levels of COVID-19 severity in the early stage of this pandemic to investigate the issues of psychological distress and protective behaviors. Specifically, they are Hong Kong general population, Taiwan healthcare workers, and Taiwan outpatients. The COVID-19 development in Hong Kong and Taiwan is described below. Since the first confirmed case on January 23, 2020, Hong Kong experienced several waves of the massive local outbreak in 2020 (30–32), given that the high population density and hence crowded living conditions, favor rapid COVID-19 transmission. The Center for Health Protection, which was established for effective disease prevention and control following the Severe Acute Respiratory Syndrome (SARS) epidemic in Hong Kong, has taken swift actions to contain the outbreak *via* comprehensive contact tracing, quarantine measures, and progressively stringent social distancing policies (33). Nonetheless, despite the good compliance to personal hygiene practices and efforts by the government and community organizations in Hong Kong, 8,847 confirmed or probable infected cases and 148 deaths were identified as of the end of December 2020 (34).

On the other hand, in Taiwan, the first case who has been confirmed having COVID-19 was announced on January 21, 2020, and nearly 1 month later (i.e., on February 16, 2020), the first death caused by COVID-19 was reported. Even after the announcement of COVID-19 pandemic (i.e., mid-March 2020), Taiwan still controlled the prevalence and infection rate of COVID-19 with satisfactory performance (35, 36). Specifically, there were only 441 confirmed cases with 7 deaths at the time of 28 May 2020 (the population size in Taiwan was over 23 million). Up to the date of the data collection period in the present study (i.e., end of December 2020), there were only 799 confirmed cases with remaining 7 deaths (7, 14).

Most Hong Kong and Taiwan citizens are originated from the Han ethnicity and therefore share similar values in Confucianism, which guide them to highly appreciate harmony, social bonding, and collectivism (37–39). Apart from the Confucianism culture, both regions have experienced the SARS community outbreak, albeit in varying severity, in 2003 (40–42). During and after the SARS community outbreak, both governments have devised guidelines on infection control (40–42). However, the demographic features are somewhat different between Hong Kong and Taiwan. Specifically, Hong Kong has a much more condensed population than Taiwan (~6,804 people per km^2^ in Hong Kong; 1,742 people per km^2^ in Taiwan). Moreover, the COVID-19 severity and development were different between Hong Kong and Taiwan. Therefore, differences in psychological distress and behavioral response are expected between Hong Kong and Taiwan.

Regarding the three populations, they may have different psychological reactions and protective behaviors toward the threat of COVID-19 (17, 43, 44). However, such comparisons in psychological distress and protective behaviors are likely to be influenced by the COVID-19 severity between studied regions across populations with different features. Specifically, the general population, healthcare workers, and outpatients may have different reactions toward the COVID-19 pandemic. Subsequently, their psychological distress, fear of COVID-19, and protective behaviors may be different. Therefore, with the data from the Hong Kong general population (who have been facing a relatively more severe COVID-19 pandemic) and those from Taiwan healthcare workers and outpatients (who resided in a less severe level of COVID-19 pandemic), we can identify the potential influence of severity of COVID-19 on different populations' psychological distress, protective behaviors, and related risk factors.

The present study thus had two aims: (i) to assess the situation of and differences in psychological distress and protective behaviors across Hong Kong general population, Taiwan healthcare workers, and Taiwan outpatients; and (ii) to investigate the potential predictors for psychological distress and protective behaviors across the populations.

## Methods

### Participants and Data Collection

A cross-sectional study design was used to recruit participants from two regions (i.e., Hong Kong and Taiwan).

Data in Hong Kong were collected from a population-wide random sample of households *via* telephone survey from 11 September to October 12, 2020. The inclusion criteria for the study were Hong Kong Chinese residents aged 18 or above. Upon successful contact with a target household, one qualified member of the household was selected among those family members using the last-birthday random selection method. Telephone interviews were carried out by experienced interviewers between 18:00 and 22:00 on weekdays. Prior appointments were arranged for suitable respondents in other periods including weekends and public holidays. Among the 12,443 dialed telephone numbers, 10,555 were invalid cases in which 254 were non-residential lines, 4,776 were fax lines/invalid lines, 1,308 were cut off immediately, and 4,217 were non-contacts after three attempts. Among the 1,888 answered calls, 28 had mid-way termination, 59 could not establish contact with an eligible person after three attempts, and 734 were refused by the eligible persons, resulting in a final sample of 1,067 respondents with a response rate of 56.5% (i.e., 1,067 respondents divided by 1,888 answered calls). This study has been approved by the Joint Chinese University of Hong Kong—New Territories East Cluster Clinical Research Ethics Committee in July 2020 with a registered number of 2,020.378.

Regarding the data collection in Taiwan, a convenience sampling was used and the survey period was between 24 September and 31 December 2020. Healthcare workers and outpatients in the National Cheng Kung University Hospital (NCKUH) were approached to complete the survey. Through the email dissemination, 500 healthcare workers and 192 outpatients from the NCKUH, which has about 5,000 employees and 1,500 beds, agreed to participate in this study. Specifically, the survey link contains clear information regarding the present study that was sent out *via* the email addresses that were stored in the NCKU Information Technology (IT) system. The first page of the online survey described the purpose and information of the study. When the participant agreed to participate, he or she should click on the agree button on this page for them to continue the online survey. The inclusion criteria for the Taiwan participants were: (i) a healthcare worker or an outpatient in the NCKUH and (ii) aged 20 years or over. The Institute Review Board (IRB) from the NCKUH approved the study with a registered number of A-ER-109-149.

### Measures

#### Psychological Distress

Depression and anxiety were the two types of distress that were used to compose the psychological distress in the present study. The 4-item Patient Health Questionnaire (PHQ-4) for Depression and Anxiety (45), which combines the Patient Health Questionnaire-2 (PHQ-2) and the Generalized Anxiety Disorder-2 (GAD-2) scales, was adopted to assess the depression and anxiety symptoms of Hong Kong participants during COVID-19. Similar four items with slightly different wordings (given that the cultures were somewhat different between Hong Kong and Taiwan) were used for Taiwan participants. All the four items were rated on a four-point Likert scale (0 = not at all; 3 = nearly every day), and the psychological distress in the present study was a standardized summated score from the four-item scores (i.e., summed scores of the four items divided by the item number of 4 to make a scale between 0 and 3). Therefore, a higher score in psychological distress indicates a higher level of distress. The internal consistency of the four items on psychological distress was excellent (Cronbach's α = 0.88). Moreover, results of the confirmatory factor analysis using maximum likelihood estimator supported the one-factor structure of the psychological distress (*p*-value of the χ^2^ = 0.23; comparative fit index = 1.000; Tucker–Lewis index = 0.999; root-mean-square-error of approximation = 0.017; and standardized root mean square residual = 0.004).

#### Protective Behaviors

Five types of behaviors were assessed according to the recommendations made by Hong Kong and Taiwan governments: regular handwashing, regular ventilation maintenance, regular household disinfection, substantial reduction of family gathering, and substantial reduction of friend gathering. A dichotomous score (yes and no) was used for assessing these protective behaviors. The internal consistency of the five items on protective behaviors was acceptable (Cronbach's α = 0.67).

#### Fear of COVID-19

One item (I am afraid of COVID-19) designed by the present authors was used to assess whether the participants feared of COVID-19. A dichotomous score (yes and no) was used for assessing fear of COVID-19.

#### Worry

Two types of worry designed by the present authors were assessed: worry on the sufficiency of personal protective equipment (PPE) and that of personal savings. A dichotomous score (yes and no) was used for assessing the two types of worry. The internal consistency of the two worry items was good (Cronbach's α = 0.73).

#### Demographic Information

Age, sex, and educational level were assessed in the survey to understand the demographics of the studied samples.

### Data Analysis

Descriptive statistics, including frequency and mean, were used to summarize the features of the studied samples. The χ^2^-tests (for categorical-dependent variables) and the independent *t*-tests (for continuous-dependent variables) were then applied to examine whether the features and demographics were significantly different between Hong Kong and Taiwan participants. Multiple linear regression models were constructed to examine predictors on psychological distress with the independent variables of age, sex, group (i.e., Hong Kong participants or Taiwan participants), fear of COVID-19, reduction of family gathering, reduction of friend gathering, worry on PPE sufficiency, and worry on personal savings. Multivariable logistic regression models were employed to examine predictors on these five types of protective behaviors with the independent variables of age, sex, group (i.e., Hong Kong participants or Taiwan participants), fear of COVID-19, worry on PPE sufficiency, and worry on personal savings. Moreover, interaction of group and sex were further investigated. Specifically, similar multiple linear regression and logistic regression models were performed with the stratification of group (by Hong Kong participants, Taiwan healthcare workers, and Taiwan outpatients) or sex (by male and female participants). For multiple linear regression models, standardized coefficients were used to present the effect size; for multivariable logistic regression models, adjusted odds ratios (AORs) were used to present the effect size. Moreover, 95% CIs of the AORs were calculated. Assumptions for multiple linear regression were checked using (i) skewness <3 and kurtosis <8 (for normality), (ii) Durbin–Watson statistics between 1.5 and 2.5 (for homoscedasticity), and (iii) variance inflation factor (VIF) value <10 (for independence). Sample size in the present study was sufficient for the constructed regression models (including multiple linear regression models and multivariable logistic regression models) according to the rule of thumb in estimating required numbers in a regression model (i.e., the ratio of subjects to predictors should range between 8 and 30) (46–50). By using the ratio of 30 and the 10 predictors, the sample size at 300 is sufficient for all the regression models in the present study. All the statistical analyses were conducted using the IBM SPSS 20.0 (IBM Corp., Armonk, NY, USA).

## Results

Table 1 presents the comparisons of the participants recruited from different areas. Specifically, the Hong Kong participants (*n* = 1,067; 68.3% aged above 50 years) were significantly older than the Taiwan healthcare workers (*n* = 500; 4.8% aged above 50 years) and Taiwan outpatients (*n* = 192; 8.9% aged above 50 years; *p* < 0.001). Regarding sex distribution, male participants were significantly more in Hong Kong participants (30.2%) and Taiwan outpatients (32.8%) than in Taiwan healthcare workers (8.0%; *p* < 0.001). In addition, the educational level in Hong Kong participants (21.0% had a degree/diploma or above) was lower than the Taiwan participants (96.6% in healthcare workers and 84.9% in outpatients had a degree/diploma or above; *p* < 0.001).

**Table 1:** Comparisons of participants' demographics between Hong Kong and Taiwan.

	***n*** **(%)**			**χ^2^ (*p*-value)**
	**Hong Kong (*n* = 1,067) General population**	**Taiwan (*n* = 500) Healthcare worker**	**Taiwan (*n* = 192) Outpatient**	
Age (in year)				681.66 (<0.001)
Below 30.00	104 (9.7)	210 (42.0)	61 (45.3)	
30.00–49.99	234 (21.9)	263 (52.6)	114 (59.4)	
50.00 or above	729 (68.3)	24 (4.8)	17 (8.9)	
Missing	0 (0.0)	3 (0.6)	0 (0.0)	
Sex				104.42 (<0.001)
Male	322 (30.2)	40 (8.0)	63 (32.8)	
Female	745 (69.8)	458 (91.6)	129 (67.2)	
Missing	0 (0.0)	2 (0.4)	0 (0.0)	
Educational level				974.94 (<0.001)
Primary or below	283 (26.5)	0 (0.0)	2 (1.0)	
Junior high	166 (15.6)	1 (0.2)	1 (0.5)	
Senior high	380 (35.6)	16 (3.2)	26 (13.5)	
Bachelor degree or diploma	205 (19.2)	441 (88.2)	120 (62.5)	
Master degree or above	19 (1.8)	38 (7.6)	43 (22.4)	
Missing	14 (1.3)	4 (0.5)	0 (0.0)	

Table 2 additionally reports the psychological distress and behavior comparisons between Hong Kong and Taiwan participants. The results indicated that Hong Kong participants had significantly lower levels of psychological distress than Taiwan participants [mean (SD) = 0.16 (0.39) vs. 0.47 (0.59) in healthcare workers and 0.46 (0.65) in outpatients; *p* < 0.001]. Moreover, Hong Kong participants (51.7%) and Taiwan outpatients had more people showing fear of COVID-19 (48.4%) than Taiwan healthcare providers (40.8%; *p* < 0.001). Hong Kong participants (93.7%) and Taiwan healthcare providers (90.8%) had more people engaging in protective behavior of handwashing than did Taiwan outpatients (60.9%; *p* < 0.001). Hong Kong participants had more people than Taiwan participants engaging in protective behaviors of maintaining ventilation (90.5 vs. 74.0% in healthcare workers and 68.8% in outpatients; *p* < 0.001); disinfecting household (78.2 vs. 39.8% in healthcare workers and 42.2% in outpatients; *p* < 0.001); reducing family gathering (39.8 vs. 7.0% in healthcare workers and 9.4% in outpatients; *p* < 0.001); and reducing friend gathering (57.2 vs. 8.2% in healthcare providers and 12.0% in outpatients; *p* < 0.001). Moreover, Hong Kong participants had less people than Taiwan participants in worrying PPE sufficiency (3.6 vs. 97.4% in healthcare providers and 16.1% in outpatients; *p* < 0.001) and personal savings (31.2 vs. 91.4% in healthcare providers and 37.5% in outpatients; *p* < 0.001).

**Table 2:** Comparisons of participants' distress and behaviors between Hong Kong and Taiwan.

	***n*** **(%)**			**χ^2^ (*p*-value)**
	**Hong Kong (*n* = 1,067) General population**	**Taiwan (*n* = 500) Healthcare worker**	**Taiwan (*n* = 192) Outpatient**	
Psychological distress^a^	0.16 (0.39)	0.47 (0.59)	0.46 (0.65)	84.52 (<0.001)
Fear of COVID-19				16.30 (<0.001)
Yes	552 (51.7)	204 (40.8)	93 (48.4)	
No	515 (48.3)	296 (59.2)	99 (51.6)	
Frequent handwashing				184.83 (<0.001)
Yes	1,000 (93.7)	454 (90.8)	117 (60.9)	
No	67 (6.3)	46 (9.2)	75 (39.1)	
Indoor ventilation				101.18 (<0.001)
Yes	966 (90.5)	370 (74.0)	132 (68.8)	
No	101 (9.5)	130 (26.0)	60 (31.2)	
Frequent disinfection				257.26 (<0.001)
Yes	834 (78.2)	199 (39.8)	81 (42.2)	
No	233 (21.8)	301 (60.2)	111 (57.8)	
Reduced family gathering				219.94 (<0.001)
Yes	425 (39.8)	35 (7.0)	18 (9.4)	
No	642 (60.2)	465 (93.0)	174 (90.6)	
Reduced friend gathering				408.69 (<0.001)
Yes	610 (57.2)	41 (8.2)	23 (12.0)	
No	457 (42.8)	459 (91.8)	169 (88.0)	
Worry about PPE sufficiency				1,410.66 (<0.001)
Yes	38 (3.6)	487 (97.4)	31 (16.1)	
No	1,029 (96.4)	13 (2.6)	161 (83.9)	
Worry about personal savings				505.00 (<0.001)
Yes	333 (31.2)	457 (91.4)	72 (37.5)	
No	734 (68.8)	43 (8.6)	120 (62.5)	

a *Presented using mean (SD) with the inferential statistics of analysis of variance. Bonferroni adjustment further indicates that Hong Kong general population had distress significantly different from the two Taiwan populations (both p-values < 0.001); the two Taiwan populations did not have significant difference in distress (p = 1.00)*.

*COVID-19, novel coronavirus disease 2019; PPE, personal protective equipment*.

The lower levels of psychological distress in Hong Kong participants than those in Taiwan participants were confirmed by the multiple linear regression when other studied variables were controlled (i.e., all the variables listed in Table 3 were entered into the same regression model) (Table 3). The assumptions for the multiple linear regression model were satisfied: skewness = 2.21, kurtosis = 5.14, Durbin–Watson statistics = 2.03, and VIF = 1.07 to 6.45. The entire multiple linear regression model was significant (*R*^2^ = 0.115; adjusted *R*^2^ = 0.110; *F*-value = 22.63; *p* < 0.001). Moreover, reduced friend gathering [reference group: No; standardized coefficient (β) = 0.074; *p* = 0.034] and worry on personal savings (reference group: No; β = 0.085; *p* = 0.003) were significantly associated with higher levels of psychological distress. However, a significant interaction of different population was found in the association between worry on personal saving and psychological distress: the relationship was positive among Hong Kong participants (β = 0.203; *p* < 0.001) and among Taiwan outpatients (β = 0.179; *p* = 0.019), but negative among Taiwan healthcare workers (β = −0.440; *p* < 0.001) (Supplementary Table S1).

**Table 3:** Multiple linear regression model in explaining psychological distress.

	**Unstandardized coefficient (SE)**	**Standardized coefficient**	***p*-value**
Age (ref: below 30.00 years)			
30.00–49.99 years	0.002 (0.032)	0.001	0.961
50.00 years or above	−0.033 (0.037)	−0.032	0.363
Sex (ref: male)	0.019 (0.028)	0.016	0.486
Group (ref: Hong Kong)			
Taiwan healthcare worker	0.268 (0.065)	0.236	<0.001
Taiwan outpatient	0.319 (0.044)	0.195	<0.001
Fear of COVID-19 (ref: no)	0.046 (0.024)	0.045	0.054
Reduce family gathering (ref: no)	0.057 (0.038)	0.050	0.128
Reduce friend gathering (ref: no)	0.077 (0.037)	0.074	0.034
Worry about PPE sufficiency (ref: no)	0.036 (0.058)	0.033	0.530
Worry about personal savings (ref: no)	0.087 (0.029)	0.085	0.003

*ref, reference group; SE, standard error; COVID-19, novel coronavirus disease 2019; PPE, personal protective equipment*.

*R^2^ = 0.115; Adjusted R^2^ = 0.110; F (p-value) = 22.63 (<0.001) for this multiple linear regression model*.

Regarding the behaviors, multivariable logistic regressions showed that female participants as compared with male participants engaged more in handwashing (AOR = 1.68; 95% CI = 1.17, 2.41), maintaining ventilation (AOR = 1.37; 95% CI = 1.00, 1.88), and disinfecting household (AOR = 1.45; 95% CI = 1.12, 1.87). Moreover, Taiwan participants as compared with Hong Kong participants engaged less in all the protective behaviors significantly (AOR = 0.02–0.68). Fear of COVID-19 was a significant predictor in all the protective behaviors (AOR = 1.36–2.61). Additionally, participants having worry on personal savings, as compared with those without worry on savings, engaged less in handwashing (AOR = 0.47; 95% CI = 0.31, 0.70), maintaining ventilation (AOR = 0.56; 95% CI = 0.40, 0.79), and disinfecting household (AOR = 0.62; 95% CI = 0.48, 0.81), but were more likely to reduce gathering with family (AOR = 2.24; 95% CI = 1.71, 2.92) and friends (AOR = 2.18; 95% CI = 1.66, 2.87) (Table 4). Stratified analyses further showed that the associations between fear of COVID-19 and personal hygiene practices, and that between worry on personal savings and all protective behaviors, were stronger in Hong Kong participants than in Taiwan participants (Supplementary Table S2).

**Table 4:** Multivariable logistic regression models in explaining protective behaviors.

	**AOR (95% CI)**				
	**Frequent handwashing**	**Indoor ventilation**	**Frequent disinfection**	**Reduce family gathering**	**Reduce friend gathering**
Age (ref: below 30 years)					
30–49.99 years	0.92 (0.60, 1.42)	1.49 (1.08, 2.06)	1.51 (1.14, 2.00)	1.21 (0.83, 1.78)	1.32 (0.92, 1.91)
50.00 years or above	0.58 (0.33, 1.02)	1.17 (0.76, 1.80)	1.33 (0.95, 1.86)	1.02 (0.70, 1.49)	1.13 (0.79, 1.62)
Sex (ref: male)	1.68 (1.17, 2.41)	1.37 (1.002, 1.88)	1.45 (1.12, 1.87)	0.93 (0.72, 1.22)	1.13 (0.87, 1.46)
Group (ref: Hong Kong)					
Taiwan healthcare worker	0.68 (0.25, 1.32)	0.44 (0.22, 0.87)	0.21 (0.12, 0.38)	0.04 (0.02, 0.08)	0.02 (0.01, 0.05)
Taiwan outpatient	0.07 (0.04, 0.12)	0.23 (0.14, 0.36)	0.21 (0.14, 0.30)	0.12 (0.07, 0.20)	0.08 (0.05, 0.13)
Fear of COVID-19 (ref: no)	2.61 (1.82, 3.74)	1.91 (1.45, 2.52)	1.36 (1.10, 1.69)	1.99 (1.57, 2.53)	1.63 (1.29, 2.05)
Worry about PPE sufficiency (ref: no)	1.23 (0.61, 2.51)	0.90 (0.49, 1.67)	1.12 (0.67, 1.89)	1.75 (0.96, 3.20)	1.76 (0.92, 3.34)
Worry about personal savings (ref: no)	0.47 (0.31, 0.70)	0.56 (0.40, 0.79)	0.62 (0.48, 0.81)	2.24 (1.71, 2.92)	2.18 (1.66, 2.87)

*AOR, adjusted odds ratio; ref, reference group; COVID-19, novel coronavirus disease 2019; PPE, personal protective equipment*.

## Discussion

The present study showed that the general population in Hong Kong experienced less psychosocial distress than the other groups in Taiwan. Although the level of fear of COVID-19 was higher in Hong Kong than in Taiwan, people in Hong Kong were less worried about the supply of PPE and their personal savings, and engaged in more protective behaviors under the pandemic. In addition to the apparent impact of reduced friend gatherings on psychosocial distress possibly due to a strong sense of collective identity in Chinese communities, further analyses showed that the association of worry about personal savings with psychosocial distress was particularly evident in Hong Kong. Moreover, being a female, fear of COVID-19 and worry about personal savings were associated with protective behaviors in general. Specifically, fear of COVID-19 was a particularly stronger facilitator of personal hygiene practices in the general population of Hong Kong compared with the other groups in Taiwan. Worrying about personal savings also tended to be a stronger risk factor of personal hygiene practices and a stronger facilitator of reduced gatherings in Hong Kong.

One interesting observation from this study is that the greater level of fear of COVID-19 in Hong Kong was accompanied by less severe psychosocial distress compared with Taiwan. The particularly stronger fear of COVID-19 in Hong Kong could plausibly be attributable to the painful experience of SARS back in 2003. Although both Hong Kong and Taiwan experienced the SARS outbreak, Hong Kong was hit more severely both in terms of infection and economic impact. Specifically, there were 1,755 SARS cases and 299 deaths (out of 6.73 million citizens) in Hong Kong (51), compared with 664 cases and 73 deaths (out of 22.6 million citizens) in Taiwan (52). In addition, the economic crisis followed by SARS was more devastating in Hong Kong with the estimated temporary shock of 2.63% gross domestic product (GDP) loss and estimated persistent shock of 3.21% GDP loss over 10 years, whereas the corresponding figures of GDP loss in Taiwan were 0.49 and 0.53% (53). Therefore, it is reasonable that the COVID-19 pandemic evoked painful memories of the SARS outbreak and hence instigated a greater level of fear in Hong Kong than in Taiwan.

However, the SARS crisis has also been turned into an opportunity in face of the COVID-19 pandemic, which has rendered a better pandemic preparedness at government and community levels, and enhanced awareness of infection control and resilience against COVID-19 distress in the general public (54–56). This echoes with our observation on the lower levels of worries and psychosocial distress in Hong Kong even though the severity of COVID-19 pandemic was greater than in Taiwan. Apart from the lesson learned from SARS, other protective factors including efforts on infection control and risk communication by the Center for Health Protection (56), strong mobilization of resources in the community (57), and the launch of social policies on financial security and employment support (58, 59) in Hong Kong could also have buffered the general public from the psychosocial distress caused by the pandemic.

In addition to the differential levels of psychosocial distress and engagement of protective behaviors, this study also identified several distinctive determinants across groups in Hong Kong and Taiwan. For example, although the fear of COVID-19 has been consistently reported as a critical predictor of positive behavioral changes (60–62), we observed a stronger association with personal hygiene practices in Hong Kong where the local COVID-19 outbreak was more severe than in Taiwan. Consistent with the Protection Motivation Theory (63), increased perceived severity of the pandemic and perceived vulnerability to COVID-19 infections could have led to the greater level of fear in densely populated Hong Kong, and hence facilitated the adoption of personal hygiene practices *via* the threat appraisal. Additionally, the previous successful experience in combating SARS *via* good hygiene practices may also have facilitated the behavioral change *via* the coping appraisal. The differential impact of fear between Hong Kong and Taiwan suggests that the association between fear of COVID-19 and personal hygiene practices may need to be induced when the pandemic severity achieves a certain level. Such an observation echoes with a recent study capturing the temporal change in COVID-19 severity in Taiwan that the association between fear and protective behaviors was not significant during March and May 2020 but became significant during May and July 2021 when the largest COVID-19 outbreak in Taiwan was emerging (64).

Another distinctive determinant between Hong Kong and Taiwan was the worry about personal savings, with its associations with distress and protective behaviors consistently more apparent in Hong Kong. The stronger impact could plausibly be explained by the greater pre-existing income inequality in Hong Kong, given that the Gini Coefficient based on post-tax post-social transfer monthly household income in 2016 was high at 0.473 in Hong Kong but only 0.336 in Taiwan (65, 66). In addition, Hong Kong has been the most unaffordable city in the world in terms of the annual cost of living, compared with a ranking of 28th for Taipei, Taiwan in 2020 (67). Taken together, those who were worried about personal savings, who also tended to be of a lower socioeconomic position in Hong Kong, may have experienced a greater level of psychosocial distress under the pandemic-related economic downturn. This is also in line with the previous COVID-19 research in Hong Kong supporting a partial mediation of inequalities in mental health *via* people's concerns over livelihood and economic activity (68, 69). Furthermore, studies in Hong Kong also revealed that the socially deprived individuals tended to be more concerned about PPE as they had lower reserves and hence utilization (68, 70), which may help explain why to worry about personal savings acted as a risk factor of personal hygiene practices but a facilitator of reduced social gatherings in Hong Kong. Adopting personal hygiene practices may be costly and so they engaged in these behaviors less frequently; instead, reducing social gatherings can help them save money and PPE.

Several limitations of this study are worth mentioning. First, due to the cross-sectional design in the surveys in Hong Kong and Taiwan, the temporal sequence of associations could not be established. Second, the residual confounding may exist in the present study's findings due to unavailable or incomparable data on health literacy, socioeconomic position, and baseline mental health status prior to COVID-19. Third, fear of COVID-19 was measured by one single item, which may not be as reliable and accurate as other more robust measurement tools, such as the Fear of COVID-19 Scale (71–74). Fourth, some measures were assessed using a dichotomous scale (i.e., protective behaviors, fear of COVID-19, and worry). Therefore, the responses in dichotomous scale may provide insufficient psychometric information for data analysis. Fifth, the measures used in the present study (except for the measure of psychological distress) were designed by the present authors. These measures have not been tested using the content validity ratio, although the present authors who are experts in this field agree that these measures are valid. Future studies should check these measures' psychometric properties if they want to use these measures. Sixth, two methods (i.e., telephone and online survey) were used for data collection. However, we are confident that the different data collection methods used in the present study do not cause serious biases, given the empirical evidence showing that different data collection methods are equivalent in the data quality (75–77). Lastly, the findings of the comparative study are generalizable only to Hong Kong and Taiwan during the period that the COVID-19 outbreak was more severe in Hong Kong, but not to other countries being harder hit by COVID-19 or to other time periods, especially when the COVID-19 situations in Hong Kong and Taiwan reversed in mid-2021. Accordingly, a large-scale study involving different parts of the world (e.g., Eastern countries and Western countries) may provide additional insightful information for worldwide policymakers to make an appropriate decision in fighting the COVID-19 pandemic.

## Conclusion

Despite the greater COVID-19 severity and fear of COVID-19 in Hong Kong, the general population in Hong Kong experienced less psychosocial distress with higher compliance to protective behaviors than the other groups in Taiwan. Worry about personal savings and fear of COVID-19 appeared to be distinctive determinants of psychosocial distress and protective behaviors in Hong Kong. Such distinctive determinants may be attributable to the differences in social contexts between Hong Kong and Taiwan, including pre-existing income inequalities, the severity of COVID-19 outbreaks, related responses at government and community levels, and the past experiences and legacy of SARS.

## Data Availability Statement

The datasets generated and analyzed during the current study are available from the corresponding author on reasonable request.

## Ethics Statement

The study was approved by the Joint Chinese University of Hong Kong—New Territories East Cluster Clinical Research Ethics Committee (registered number: 2020.378) and the Institute Review Board from the National Cheng Kung University Hospital (registered number: A-ER-109-149). The study was performed in accordance with the ethical standards laid down in the 1964 Declaration of Helsinki and its later amendments.

## Author Contributions

C-YL and GC performed material preparation, data collection, data analyses, and written the first draft of the manuscript. All authors contributed to conception and design of the study, contributed to data interpretation, critically commented on previous versions of the manuscript, and read and approved the final manuscript.

## Funding

This study was supported in part by the Ministry of Science and Technology, Taiwan (Grant No. MOST109-2327-B-006-005), in part by the Taipei Municipal Wanfang Hospital Cross-Institutions Fund (Grant No. 110-swf-01), and in part by the Higher Education Sprout Project, Ministry of Education to the Headquarters of University Advancement at National Cheng Kung University (NCKU). Data collection in Hong Kong was also supported by a research project grant from the Chinese University of Hong Kong Institute of Health Equity, which was funded by the Vice-Chancellor's Discretionary Fund of the Chinese University of Hong Kong (Project Ref No.: 136604080).

## Conflict of Interest

The authors declare that the research was conducted in the absence of any commercial or financial relationships that could be construed as a potential conflict of interest.

## Publisher's Note

All claims expressed in this article are solely those of the authors and do not necessarily represent those of their affiliated organizations, or those of the publisher, the editors and the reviewers. Any product that may be evaluated in this article, or claim that may be made by its manufacturer, is not guaranteed or endorsed by the publisher.
