# Supplementary material for: Psychological Distress and Protective Behaviors During the COVID-19 Pandemic Among Different Populations: Hong Kong General Population, Taiwan Healthcare Workers, and Taiwan Outpatients
Source: Front Med (Lausanne). 2022 Feb 15;9:800962. doi: 10.3389/fmed.2022.800962 (PMC8885588; doi:10.3389/fmed.2022.800962)
Supplement: Supplementary file 1 [file Table_1.docx]

| **Supplementary Table S1. Multivariable linear regression models in explaining distress, stratified by data source** | | | | | | | | | | |  |  |
| --- | --- | --- | --- | --- | --- | --- | --- | --- | --- | --- | --- | --- |
|  |  | HK |  |  |  | TW_hcw |  |  |  | TW_pt |  |  |
|  |  | Unstandardized beta (SE) | Standardized beta | p |  | Unstandardized beta (SE) | Standardized beta | p |  | Unstandardized beta (SE) | Standardized beta | p |
|  | Age (Ref: below 30.00 years) |  |  |  |  |  |  |  |  |  |  |  |
|  | 30.00-49.99 years | -0.070 (0.045) | -0.074 | 0.124 |  | 0.012 (0.054) | 0.010 | 0.831 |  | 0.155 (0.097) | 0.118 | 0.111 |
|  | 50.00 years or above | -0.117 (0.040) | -0.139 | 0.004 |  | 0.268 (0.125) | 0.097 | 0.032 |  | 0.228 (0.166) | 0.101 | 0.171 |
|  | Gender (Ref: Male) | 0.015 (0.026) | 0.017 | 0.563 |  | 0.051 (0.087) | 0.026 | 0.559 |  | 0.056 (0.094) | 0.040 | 0.554 |
|  | Fear of COVID-19 (Ref: No) | 0.031 (0.025) | 0.039 | 0.213 |  | 0.068 (0.053) | 0.057 | 0.197 |  | 0.003 (0.090) | 0.003 | 0.969 |
|  | Reduced family gathering  (Ref: No) | 0.031 (0.032) | 0.039 | 0.332 |  | 0.298 (0.152) | 0.128 | 0.052 |  | -0.399 (0.306) | -0.181 | 0.194 |
|  | Reduced friend gathering  (Ref: No) | 0.028 (0.031) | 0.036 | 0.360 |  | 0.056 (0.142) | 0.026 | 0.695 |  | 0.893 (0.276) | 0.450 | 0.001 |
|  | Worry about PPE sufficiency (Ref: No) | -0.085 (0.065) | -0.040 | 0.193 |  | -0.020 (0.166) | -0.005 | 0.904 |  | 0.209 (0.133) | 0.119 | 0.117 |
|  | Worry about personal savings (Ref: No) | 0.172 (0.027) | 0.203 | <0.001 |  | -0.440 (0.094) | -0.209 | <0.001 |  | 0.238 (0.101) | 0.179 | 0.019 |

HK=Hong Kong; TW=Taiwan; hcw=healthcare workers; pt=patient; SE=standard error; Ref=reference group; COVID-19=novel coronavirus disease 2019; PPE=personal protective equipment.
