# Supplementary material for: Psychological Distress and Protective Behaviors During the COVID-19 Pandemic Among Different Populations: Hong Kong General Population, Taiwan Healthcare Workers, and Taiwan Outpatients
Source: Front Med (Lausanne). 2022 Feb 15;9:800962. doi: 10.3389/fmed.2022.800962 (PMC8885588; doi:10.3389/fmed.2022.800962)
Supplement: Supplementary file 2 [file Table_2.docx]

| **Supplementary Table S2. Multivariable logistic regression models in explaining behaviors, stratified by data source** | | | | | | |
| --- | --- | --- | --- | --- | --- | --- |
|  |  | HK |  | TW_hcw |  | TW_pt |
|  |  | AOR (95% CI) |  | AOR (95% CI) |  | AOR (95% CI) |
| ***Frequent handwashing*** | |  |  |  |  |  |
|  | Age (Ref: below 30.00 years) |  |  |  |  |  |
|  | 30.00-49.99 years | 1.10 (0.31, 3.84) |  | 0.69 (0.36, 1.32) |  | 1.15 (0.59, 2.26) |
|  | 50.00 years or above | 0.52 (0.18, 1.49) |  | 1.71 (0.22, 13.59) |  | 0.85 (0.27, 2.66) |
|  | Gender (Ref: Male) | 1.75 (1.04, 2.90)* |  | 0.66 (0.20, 2.12) |  | 2.09 (1.11, 3.95)* |
|  | Fear of COVID-19 (Ref: No) | 5.57 (2.89, 10.74)*** |  | 1.59 (0.82, 3.08) |  | 1.78 (0.95, 3.32) |
|  | Worry about PPE sufficiency (Ref: No) | 0.68 (0.18, 2.55) |  | 0.68 (0.08, 5.70) |  | 1.62 (0.64, 4.08) |
|  | Worry about personal savings (Ref: No) | 0.35 (0.20, 0.60)*** |  | 1.73 (0.67, 4.50) |  | 0.41 (0.20, 0.83)* |
| ***Indoor ventilation*** | |  |  |  |  |  |
|  | Age (Ref: below 30.00 years) |  |  |  |  |  |
|  | 30.00-49.99 years | 1.97 (0.81, 4.82) |  | 1.52 (1.01, 2.29)* |  | 1.16 (0.59, 2.29) |
|  | 50.00 years or above | 1.02 (0.49, 2.09) |  | 4.81 (1.10, 21.14)* |  | 2.35 (0.59, 9.26) |
|  | Gender (Ref. Male) | 1.30 (0.83, 2.03) |  | 0.96 (0.48, 1.91) |  | 1.75 (0.92, 3.35) |
|  | Fear of COVID-19 (Ref: No) | 5.19 (3.07, 8.76)*** |  | 1.11 (0.73, 1.68) |  | 1.21 (0.64, 2.30) |
|  | Worry about PPE sufficiency (Ref: No) | 0.39 (0.15, 1.03) |  | 0.40 (0.08, 1.91) |  | 1.46 (0.56, 3.81) |
|  | Worry about personal savings (Ref: No) | 0.31 (0.20, 0.50)*** |  | 1.54 (0.77, 3.09) |  | 0.65 (0.32, 1.33) |
| ***Frequent disinfection*** | |  |  |  |  |  |
|  | Age (Ref: below 30.00 years) |  |  |  |  |  |
|  | 30.00-49.99 years | 1.99 (1.13, 3.51)* |  | 1.37 (0.94, 2.00) |  | 1.12 (0.58, 2.16) |
|  | 50.00 years or above | 1.30 (0.81, 2.09) |  | 2.64 (1.12, 6.27)* |  | 2.72 (0.87, 8.53) |
|  | Gender (Ref. Male) | 1.72 (1.27, 2.35)*** |  | 0.75 (0.41, 1.37) |  | 1.27 (0.67, 2.41) |
|  | Fear of COVID-19 (Ref: No) | 1.70 (1.25, 2.32)** |  | 0.90 (0.62, 1.31) |  | 1.59 (0.87, 2.91) |
|  | Worry about PPE sufficiency (Ref: No) | 0.86 (0.39, 1.87) |  | 2.14 (0.56, 8.15) |  | 1.01 (0.40, 2.51) |
|  | Worry about personal savings (Ref: No) | 0.54 (0.39, 0.78)*** |  | 0.91 (0.47, 1.78) |  | 0.56 (0.28, 1.11) |
| ***Reduced family gathering*** | |  |  |  |  |  |
|  | Age (Ref: below 30.00 years) |  |  |  |  |  |
|  | 30.00-49.99 years | 1.75 (1.05, 2.92)* |  | 0.59 (0.29, 1.23) |  | 0.72 (0.25, 2.10) |
|  | 50.00 years or above | 1.32 (0.83, 2.09) |  | 1.49 (0.40, 5.52) |  | 0.42 (0.05, 3.79) |
|  | Gender (Ref. Male) | 0.90 (0.68, 1.20) |  | 1.05 (0.32, 3.44) |  | 1.14 (0.38, 3.44) |
|  | Fear of COVID-19 (Ref: No) | 2.09 (1.60, 2.72)*** |  | 1.04 (0.52, 2.11) |  | 3.02 (1.003, 9.07)* |
|  | Worry about PPE sufficiency (Ref: No) | 2.17 (1.01, 4.68)* |  | NA ^a^ |  | 0.86 (0.19, 3.81) |
|  | Worry about personal savings (Ref: No) | 2.35 (1.77, 3.12)*** |  | 0.81 (0.23, 2.83) |  | 1.14 (0.38, 3.43) |
| ***Reduced friend gathering*** | |  |  |  |  |  |
|  | Age (Ref: below 30.00 years) |  |  |  |  |  |
|  | 30.00-49.99 years | 1.52 (0.93, 2.47) |  | 1.16 (0.59, 2.31) |  | 0.72 (0.27, 1.89) |
|  | 50.00 years or above | 1.20 (0.78, 1.84) |  | 2.52 (0.76, 8.39) |  | 0.71 (0.13, 3.79) |
|  | Gender (Ref. Male) | 1.11 (0.84, 1.47) |  | 0.93 (0.33, 2.64) |  | 1.67 (0.58, 4.81) |
|  | Fear of COVID-19 (Ref: No) | 1.69 (1.30, 2.18)*** |  | 0.92 (0.47, 1.78) |  | 2.63 (1.001, 6.91)* |
|  | Worry about PPE sufficiency (Ref: No) | 2.05 (0.83, 5.10) |  | NA ^a^ |  | 1.40 (0.40, 4.93) |
|  | Worry about personal savings (Ref: No) | 2.37 (1.76, 3.19)*** |  | 1.00 (0.29, 3.45) |  | 0.95 (0.34, 2.63) |
| ^a^ None of those who were not worried about PPE sufficiency reduced family and friend gatherings a lot  HK=Hong Kong; TW=Taiwan; hcw=healthcare workers; pt=patient; AOR=adjusted odds ratio; CI=confidence interval; Ref=reference group; COVID-19= novel coronavirus disease 2019; PPE=personal protective equipment. | | | | | | |
| * p<0.05; ** p<0.01; *** p<0.001 | | | | | | |
